# Concordance between Self-Reports and Medicare Claims among Participants in a National Study of Chronic Disease Self-Management Program

**DOI:** 10.3389/fpubh.2015.00222

**Published:** 2015-10-08

**Authors:** Luohua Jiang, Ben Zhang, Matthew Lee Smith, Andrea L. Lorden, Tiffany A. Radcliff, Kate Lorig, Benjamin L. Howell, Nancy Whitelaw, Marcia G. Ory

**Affiliations:** ^1^Department of Epidemiology, School of Medicine, University of California Irvine, Irvine, CA, USA; ^2^Department of Epidemiology and Biostatistics, School of Public Health, Texas A&M Health Science Center, College Station, TX, USA; ^3^Department of Health Promotion and Behavior, College of Public Health, University of Georgia, Athens, GA, USA; ^4^Department of Health Policy and Management, School of Public Health, Texas A&M Health Science Center, College Station, TX, USA; ^5^Division of Immunology and Rheumatology, Department of Medicine, Stanford University, Stanford, CA, USA; ^6^CVS Health, Baltimore, MD, USA; ^7^Center for Healthy Aging, National Council on Aging, Washington, DC, USA; ^8^Department of Health Promotion and Community Health Sciences, School of Public Health, Texas A&M Health Science Center, College Station, TX, USA

**Keywords:** aging, chronic disease, claims data, disease management, health services

## Abstract

**Objectives:**

To evaluate the concordance between self-reported data and variables obtained from Medicare administrative data in terms of chronic conditions and health care utilization.

**Design:**

Retrospective observational study.

**Participants:**

We analyzed data from a sample of Medicare beneficiaries who were part of the National Study of Chronic Disease Self-Management Program (CDSMP) and were eligible for the Centers for Medicare and Medicaid Services (CMS) pilot evaluation of CDSMP (*n* = 119).

**Methods:**

Self-reported and Medicare claims-based chronic conditions and health care utilization were examined. Percent of consistent numbers, kappa statistic (κ), and Pearson’s correlation coefficient were used to evaluate concordance.

**Results:**

The two data sources had substantial agreement for diabetes and chronic obstructive pulmonary disease (COPD) (κ = 0.75 and κ = 0.60, respectively), moderate agreement for cancer and heart disease (κ = 0.50 and κ = 0.47, respectively), and fair agreement for depression (κ = 0.26). With respect to health care utilization, the two data sources had almost perfect or substantial concordance for number of hospitalizations (κ = 0.69–0.79), moderate concordance for ED care utilization (κ = 0.45–0.61), and generally low agreement for number of physician visits (κ ≤ 0.31).

**Conclusion:**

Either self-reports or claim-based administrative data for diabetes, COPD, and hospitalizations can be used to analyze Medicare beneficiaries in the US. Yet, caution must be taken when only one data source is available for other types of chronic conditions and health care utilization.

## Introduction

Chronic conditions and health care utilization are important measurements in health interventions and other health-related studies. These measures may be captured either by patient self-reports or through some type of administrative data. Both types of data have advantages and limitations. Self-report data are cost efficient and inclusive of all sources of health care, but suffer from recall bias and inaccuracy. Conversely, claim-based administrative data could be more objective and accurate but are limited by coding errors as well as its inability to cover out-of-plan use.

Although investigated by multiple research groups in the past (1–19), the concordance between self-reports and administrative data is not well established. The accuracy of self-reported chronic conditions has been found to vary for different conditions (1, 4, 9, 11–16). Diabetes usually has substantial to almost perfect agreement between self-reported status and diagnosis from medical records or claim-based data (4, 9, 11–16). Yet, the validity of self-reports for other chronic conditions was not as high. For example, fair to moderate agreement between self-reports and claim-based data has been reported for heart disease in both US veterans (11) and older adults (13) as well as a national sample of Taiwanese (12). The concordance of two different data sources for mental disorders such as depression is usually fair or slight in various settings (4, 11, 12, 15). For chronic obstructive pulmonary disease (COPD), previous reports have been inconsistent in the literature. Some studies found moderate to substantial concordance between self-reported and administrative data among older emergency department patients (5) and hospitalized patients (16). But other studies only found fair agreement in a large sample of the general population in ON, Canada (9), in the US Veterans Affairs health care setting (11), and among participants of the 2005 Taiwan National Health Interview Survey (12).

**Figure 1:**
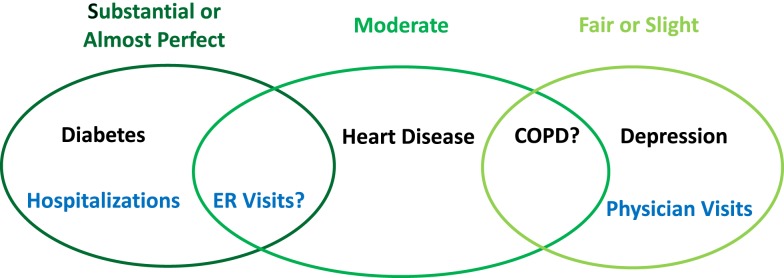
**Conceptual framework for concordance between self-reported and administrative data**.

With respect to health care utilization, some researchers reported that self-reports and administrative claims match better when the health event is more salient to the individual (2, 3, 6, 12, 13). For instance, most previous studies reported substantial agreement between self-reported hospitalizations and inpatient stay identified from administrative data (2, 3, 6, 12, 13, 17). A few studies investigated the concordance for emergency room visits between two different data resources and found generally good agreement, but the degree of agreement was less than that for hospitalizations in the same study (3, 12). Conversely, the agreement for outpatient physician visits was usually low (2, 3, 6, 13). A recent study reported that age and aging had an important effect on the concordance – older participants tended to have lower rate of concordant reporting (13). We have summarized the existing evidence and our hypotheses for the concordance of different chronic conditions and health care utilization as a conceptual framework shown in Figure 1.

In this study, we aim to investigate the concordance issue among Medicare beneficiaries participating in an evidence-based chronic disease management program, of which the participants were older adults with at least one common chronic condition. Specifically, the pilot evaluation of the Chronic Disease Self-Management Program (CDSMP) examined the impact of the CDSMP on health care utilization and costs in a sample of Medicare beneficiaries who are part of the National Study of CDSMP (20, 21). This pilot evaluation was also designed to inform Centers for Medicare and Medicaid Services (CMS) about the concordance between self-reported data collected by the study questionnaires and corresponding measurements identified using Medicare administrative data.

## Materials and Methods

### Participants

The CMS pilot evaluation of CDSMP was designed as a retrospective observational study of adults enrolled in an evidence-based self-management program for chronic disease. The Stanford CDSMP aids individuals with chronic diseases to develop self-management skills that improve health status through an evidence-based disease prevention model in community-based settings. CDSMP workshops were delivered throughout the US by 22 licensed sites in 17 states that enrolled respondents between August 2010 and April 2011 (21). Written informed consent was obtained from 1,170 individuals to collect and use survey data about health status, health care utilization, and other self-reported health care measures relevant to a participant’s chronic conditions. Part of the survey data included the participant’s name, mailing address, state, ZIP code, birth date, and gender.

The pilot evaluation study was based on a subset of CDSMP respondents who (1) were at least age 65.5 years at the beginning of the National Study of CDSMP; (2) reported having Medicare in their CDSMP survey responses; (3) actively consented to the CMS study (i.e., agreed to have their self-reported survey data linked to Medicare Administrative Data); and (4) did not have Health Maintenance Organization (HMO) enrollment in the 18 months before the CDSMP class start date. HMO enrollees were excluded because most Medicare Advantage payments would not be captured in the study datasets. Among the 1,170 CDSMP participants, 676 of them were 65.5 years or older at the beginning of the program and reported having Medicare. However, only 267 of them consented to participate in the CMS pilot evaluation.

Only those ZIP Codes that housed either a CDSMP workshop or a consented participant’s residence were identified for Medicare administrative data extracts. The details of the linking process are described elsewhere (22). Briefly, the Medicare Vital Status File with names and addresses was used to identify the correct beneficiary identification number (BIN) for each consented participant. The variables in the Vital Status File relevant to linking CDSMP participants to their Medicare utilization data included beneficiary name, beneficiary date of birth, beneficiary gender, beneficiary mailing address, beneficiary state, and beneficiary ZIP code. Following a block-based fuzzy matching algorithm, we successfully linked 208 CDSMP participants to their Medicare administrative data, representing a 78% linkage rate (out of 267 consented participants). Among these 208 linked individuals, 89 of them were excluded because they had HMO enrollment in the 18 months before the CDSMP class start date. Thus, the final sample size of the current study is 119.

The pilot evaluation was carried out in accordance with the recommendations by the IRBs of Stanford University and Texas A&M University with written informed consent from all subjects.

### Data sources

#### Self-Reported Data

Participants of the National Study of CDSMP completed questionnaires at three time points: baseline, 6 months, and 12 months after program enrollment. Self-reported questionnaires collected information about participants’ demographic characteristics, chronic conditions, health-related behaviors, and health care utilization.

At baseline, the CDSMP participants were asked to report the number and type of chronic conditions with which they had been diagnosed. The survey question was “Please indicate below which chronic condition(s) you have (check all that apply).” The response options included type 2 diabetes; type 1 diabetes; asthma; chronic bronchitis, emphysema, or COPD; other lung disease; high blood pressure; heart disease; arthritis or other rheumatic disease; cancer, depression; anxiety or other emotional/mental health condition; and other chronic condition. Depression was also measured using the PHQ-8, where a PHQ-8 score of 10 or higher is defined as depression (23). Health care utilization was measured by a series of self-reported items, asking respondents to indicate the number of non-emergency physician visits they had (physician visits), number of emergency room visits (ER visits), and number of times hospitalized for one night or longer (hospital stays) in the past 6 months.

#### Claims-Based Data

Variables from several Medicare administrative data files were requested. These included the Vital Status File, Beneficiary Annual Summary Files (BASF), Medicare fee-for-service institutional claim summary and revenue line data files, Medicare fee-for-service non-institutional claim summary and claim line data files, the hierarchal condition category (HCC) concurrent risk scores and indicators, and MedPAR data. Beneficiary unique identifiers were then linked across other datasets to select the relevant data for the concordance analysis of the current study.

Claims-based chronic conditions were identified through HCC chronic condition indicators (24). Specifically, variables hcc_15_cd to hcc_19_cd and hirchcl_15_cd to hirchcl_18_cd were used to identify those with diabetes; hcc_108_cd was used to identify those with COPD; hcc_80_cd to hcc_83_cd, hcc_92_cd, and hirchcl_81_cd, hirchcl_82_cd were used for heart disease identification; and hcc_55_cd was used to identify depression. To identify physician visits, the non-institutional physician/supplier data file was used to find all claims by the following combinations of BETOS codes and HCPCS codes: M1A: 99201–99205 or M1B: 99211–99215. The number of physician office visits was then calculated by counting the number of unique claim-from-date values from the claims identified. Outpatient events were identified from institutional claims data by counting the number of unique claim-from-date values for each beneficiary among the claims with a NCH claim type code of 40, excluding outpatient ER utilization. ER utilization was calculated by adding the number of unique claim-from-date values from institutional claims line files using the revenue center code values of 0450–0459 and 0981. Inpatient stays were identified by counting the number of unique claim-from-date values among the claims with a NCH claim type code of 60 or 61 in institutional claims data. Claims for those beneficiaries seen in the ER and admitted to the hospital were counted as inpatient stays only, not ER care utilization.

### Statistical analyses

Concordance for various chronic conditions was evaluated using kappa statistic (κ). In addition, because the magnitude of kappa statistic is highly influenced by the prevalence of the condition as well as the bias between the two data sources, we also reported several other values, including the bias index (BI), prevalence index (PI), and the prevalence-adjusted bias-adjusted kappa (PABAK). The BI ranges from 0 to 1, with 0 indicating no bias and 1 implying that one data source never identifies the condition while the other source always does. The PI also ranges from 0 to 1, with 0 indicating the prevalence of the condition is 50%, while 1 suggesting the prevalence of the condition is 0 or 100%. PABAK reflects the concordance under a hypothetically ideal situation, where no prevalence or bias effects exist. On its own, PABAK is uninformative. It should always be presented along with kappa statistic, to inform the readers about the possible effects of prevalence and bias (25, 26). For health care utilization variables, the following steps were taken to assess the concordance between self-reports and claims data: (1) calculate the percent of participants with the same number of health care utilization in the same time period from the two data sources; (2) calculate rates of concordance by considering a discrepancy of ±1 visit as concordance; (3) calculate the Pearson’s correlation coefficient between the number of utilization from two sources; (4) dichotomize each utilization variable to a yes/no variable for any utilization in the past 6 months and then estimate kappa statistic, BI, PI, and PABAK to assess the degree of agreement between the dichotomized utilization variables.

## Results

### Sample characteristics

In total, 267 CDSMP participants (39% of all potentially eligible participants) consented to the pilot evaluation. Table 1 illustrates the average age, gender composition, race distribution, and years of education were not significantly different between those who consented and those who did not consent. However, the consented respondents had significantly more physician visits (3.78 vs. 3.09, *P* = 0.01) and more hospitalizations (0.21 vs. 0.14, *P* = 0.03) at baseline than the respondents who did not consent. Furthermore, the consented respondents had significantly more co-morbidities (3.21 vs. 2.78, *P* = 0.0004) and higher rate of diabetes (38.6 vs. 27.1%, *P* = 0.002).

**Table 1:** **Participant baseline characteristics**.

	Potentially eligible participants			Linked participants		
	Consented	Not consented	*P*-value*	No HMO	Some HMO	*P*-value*
	*N* (%)^a^	*N* (%)^a^		*N* (%)^a^	*N* (%)^a^	
Number of participants	267	409		119	89	
**Demographic characteristics**
Average age in years (mean ± SD)	75.8 (±7.0)	75.2 (±6.6)	0.25	75.3 (±6.6)	74.1 (±6.6)	0.19
Female	222 (83.2)	341 (83.4)	0.94	99 (83.2)	76 (85.4)	0.67
Race/ethnicity			0.16			0.13
Latino/Hispanic	49 (18.4)	64 (15.7)		15 (12.6)	14 (15.7)	
Non-hispanic white	166 (62.2)	245 (60.2)		85 (71.4)	53 (59.6)	
African American	40 (15.0)	56 (13.8)		17 (14.3)	15 (12.6)	
Asian/Pacific Islander	6 (2.3)	24 (5.9)		0 (0.0)	4 (4.5)	
American Indian/Alaska Native	1 (0.4)	3 (0.7)		0 (0.0)	1 (1.1)	
Other	5 (1.9)	15 (3.7)		2 (1.7)	2 (2.3)	
Average years of education (from 1 to 23)	13.1 (±3.9)	12.9 (±3.6)	0.52	13.8 (±3.3)	12.8 (±3.9)	0.05
**Health care utilization**
Number of physician visits (mean ± SD)	3.78 (±3.52)	3.09 (±3.08)	0.01	4.34 (±4.40)	3.33 (±2.30)	0.03
Number of emergency room visits (mean ± SD)	0.16 (±0.45)	0.18 (±0.13)	0.64^§^	0.22 (±0.54)	0.16 (±0.42)	0.56^§^
Number of hospitalizations (mean ± SD)	0.21 (±0.51)	0.14 (±0.47)	0.03^§^	0.25 (±0.59)	0.18 (±0.44)	0.56^§^
**Chronic conditions**
Number of co-morbidities (mean ± SD)	3.21 (±1.69)	2.78 (±1.53)	0.0004	3.32 (±1.68)	2.96 (±1.62)	0.15
Diabetes	103 (38.6)	111 (27.1)	0.002	38 (31.9)	39 (43.8)	0.08
Depression	62 (23.2)	79 (19.3)	0.22	27 (22.7)	22 (24.7)	0.73
Heart disease	66 (24.7)	95 (23.2)	0.66	34 (28.6)	16 (18.0)	0.08
COPD	68 (25.5)	79 (19.3)	0.06	40 (33.6)	19 (21.4)	0.05

*^a^Unless otherwise specified*.

*P-values of chi-square tests for categorical variables and independent sample *t*-tests for continuous variables comparing consented and not-consented CDSMP participants.

*^§^*P*-values of Wilcoxon rank sum tests*.

Among the 208 CDSMP participants who were linked to their available Medicare administrative data, 119 had no HMO coverage and were eligible for study analyses. The linked participants with and without HMO coverage did not differ significantly for most of the characteristics. Yet, the participants without HMO coverage had more years of education (13.8 vs. 12.8, *p* = 0.05) and had more baseline physician visits (4.34 vs. 3.33, *p* = 0.03) than those who had some HMO coverage.

### Chronic disease status

Table 2 presents the concordance analysis results for various chronic conditions. At baseline, the two data sources had substantial agreement for diabetes and COPD status. The kappa statistics for the agreement between the two data sources for these two conditions were 0.75 for diabetes and 0.60 for COPD. Self-reports and Medicare administrative data had moderate agreement for heart disease, with a kappa statistic of 0.47. All these conditions had very small BI (<0.03), suggesting similar prevalence rates of the two data sources. Although they all had relatively higher PI, the values of PABAK were similar to slightly higher than the corresponding kappa statistic.

**Table 2:** **Concordance analysis for chronic conditions at baseline**.

	Status from Medicare administrative data				κ	|BI|	|PI|	PABAK
	*N* (%)	No	Yes	Total	
**Diabetes**					0.75	0.03	0.37	0.78
Self-reported status	No	75 (63.0)	8 (6.7)	83 (69.8)				
	Yes	5 (4.2)	31 (26.1)	36 (30.3)				
	Total	80 (67.2)	39 (32.8)	119 (100)				
**Chronic obstructive pulmonary disease (COPD)**					0.60	0.03	0.58	0.73
Self-reported status	No	86 (72.3)	6 (5.0)	92 (77.3)				
	Yes	10 (8.4)	17 (14.3)	27 (22.7)				
	Total	96 (80.7)	23 (19.3)	119 (100)				
**Heart disease**					0.47	0.03	0.45	0.58
Self-reported status	No	74 (62.2)	11 (9.2)	85 (71.4)				
	Yes	14 (11.8)	20 (16.8)	34 (28.6)				
	Total	88 (74.0)	31 (26.1)	119 (100)				
**Depression**					0.26	0.18	0.73	0.63
Self-reported status	No	92 (77.3)	0 (0.0)	92 (77.3)				
	Yes	22 (18.5)	5 (4.2)	27 (22.7)				
	Total	114 (95.8)	5 (4.2)	119 (100)				
**Depression**					0.11	0.12	0.79	0.65
Status determined by PHQ_8	No	96 (81.4)	3 (2.5)	99 (83.9)				
	Yes	17 (14.4)	2 (1.7)	19 (16.1)				
	Total	113 (95.8)	5 (4.2)	118 (100)				

	**Self-reported status**							
	***N* (%)**	**No**	**Yes**	**Total**				

**Depression**					0.20	0.07	0.61	0.48
Status determined by PHQ_8	No	80 (67.8)	19 (16.1)	99 (83.9)				
	Yes	11 (9.3)	8 (6.8)	19 (16.1)				
	Total	91 (77.1)	27 (22.9)	118 (100)				

The two data sources had fair agreement for depression. All of the 22 inconsistent participants self-reported having depression at baseline, but no depression or bi-polar disorder-related claim was identified in the Medicare administrative data in 2009 or 2010. The kappa statistics for this variable was 0.26. Additionally, the depression status determined by PHQ-8 only had slight agreement with either self-reported depression (κ = 0.20) or Medicare administrative data (κ = 0.11). The BI for depression was between 0.11 and 0.26, whereas the PI for this condition ranges from 0.61 to 0.79. In this case, PABAK (0.48–0.65) was substantially higher than their corresponding kappa statistic (0.11–0.26).

### Health care utilization

From the Medicare administrative data, two variables related to physician encounters were identified: (1) the number of outpatient visits from the Institutional claims files and (2) the number of physician office visits from the non-Institutional claims files. As shown in Table 3, neither of these two variables had good concordance with self-reported physician visits, even when a difference of ±1 between two variables is considered concordant. Except number of outpatient visits, the other two situations had very low BI (≤0.1). All three measures had very high PI, which led to substantially higher PABAK than the corresponding kappa statistic.

**Table 3:** **Summary of concordance analysis for health care utilization**.

	Self-report, mean (SD)	Medicare data, mean (SD)	Consistent number (%)	Consistent (***±***1) number (%)	Pearson’s correlation coefficient	*κ*^b^	|BI|	|PI|	PABAK
**Number of outpatient utilization^a^**
Baseline	4.34 (4.40)	0.39 (1.14)	13 (10.9)	32 (26.9)	0.25	0.06	0.26	0.69	0.45
6 months	4.43 (4.30)	0.44 (1.18)	21 (18.6)	38 (33.6)	0.10	0.11	0.18	0.72	0.54
12 months	4.31 (4.03)	0.35 (1.61)	12 (11.4)	29 (27.6)	0.07	0.01	0.27	0.68	0.39
**Number of physician visits^a^**
Baseline	4.34 (4.40)	5.41 (4.11)	11 (9.2)	43 (36.1)	0.39	0.26	0.07	0.88	0.83
6 months	4.43 (4.30)	5.32 (4.28)	17 (15.0)	44 (38.9)	0.45	0.24	0.07	0.82	0.75
12 months	4.31 (4.03)	4.97 (3.88)	12 (11.4)	31 (29.5)	0.38	0.08	0.10	0.85	0.73
**Number of physician and outpatient utilization^a^**
Baseline	4.34 (4.40)	5.58 (4.31)	8 (6.7)	20 (16.8)	0.36	0.15	0.04	0.91	0.85
6 months	4.43 (4.30)	5.44 (4.35)	14 (12.4)	26 (23.0)	0.33	0.31	0.04	0.85	0.81
12 months	4.31 (4.03)	5.11 (4.01)	7 (6.7)	19 (18.1)	0.24	0.11	0.07	0.88	0.79
**Number of ER visits**
Baseline	0.22 (0.54)	0.32 (1.02)	98 (82.4)	107 (89.9)	0.29	0.45	0.03	0.71	0.73
6 months	0.17 (0.48)	0.40 (1.14)	93 (82.3)	102 (90.3)	0.53	0.61	0.03	0.71	0.81
12 months	0.20 (0.49)	0.29 (1.29)	88 (83.8)	99 (94.3)	0.52	0.51	0.06	0.73	0.77
**Number of inpatient stays**
Baseline	0.25 (0.59)	0.17 (0.49)	107 (89.9)	115 (96.6)	0.67	0.75	0.05	0.68	0.87
6 months	0.19 (0.50)	0.12 (0.35)	102 (90.3)	111 (98.2)	0.64	0.69	0.05	0.73	0.86
12 months	0.19 (0.50)	0.12 (0.35)	99 (94.3)	104 (99.1)	0.79	0.79	0.03	0.74	0.90

*^a^Compared with the number of self-reported physician visits in baseline CDSMP survey data*.

*^b^Kappa statistics were calculated based on any utilization or visit*.

Self-reports and Medicare administrative data had moderate agreement with respect to ER utilization. Specifically, 82% or more linked CDSMP participants had the same number of ER visits in both data sources at each time point. When comparing the dichotomized status of ER visits (yes/no for any ER visits), the kappa statistics for the agreement of the two data sources was 0.45 at baseline, 0.61 at 6-month, and 0.51 at 12-month follow-up. A high percentage (i.e., 89.9, 90.3, and 94.3%) of the linked participants had the same number of hospitalizations in both data sources at the three time points. When comparing the dichotomized status of inpatient visits (yes/no for any hospitalization), the kappa statistics for the agreement of the two data sources was 0.75, 0.69, and 0.79 at baseline, 6-month, and 12-month, respectively, indicating substantial agreement.

The BI for ER visits and hospitalizations was small (0.03–0.06), but the PI for them was relatively large (0.68–0.74). The PABAKs for both ER utilizations and inpatient visits were substantially higher than their corresponding kappa statistics.

## Discussion

The results of this study are consistent with previous reports in finding substantial agreement for diabetes, moderate agreement for heart disease, and fair agreement for depression (1, 4, 9, 11–16). The results for depression may be a reflection of the under-diagnosis for mental illness among Medicare beneficiaries which was partially caused by the beneficiaries’ unwillingness to seek treatment (27). Neither the self-reported status nor the claim-based depression diagnosis had a good agreement with the PHQ-8-based depression diagnosis, suggesting investigators must be cautious in depression-related analysis when only one data source for depression is available. The concordance for COPD between self-reports and medical record/administrative data has been inconsistent in the literature. Some studies found moderate to substantial concordance (5, 16), but others only found fair agreement (9, 11, 12). The results of the current study add to the body of evidence about the potential accuracy of using self-reports or Medicare claims data to identify this condition among Medicare beneficiaries interested in evidence-based chronic disease management program.

Regarding health care utilization, the two data sources had almost perfect or substantial concordance for number of hospitalizations, and moderate concordance for ER utilization. Yet, the self-reported number of physician visits had generally low agreement with the potentially corresponding physician visits variables available in the Medicare administrative data. These findings are also consistent with previous studies (2, 3, 6, 12, 13, 17). In an earlier study of 216 CDSMP participants who received care through Kaiser Permanente HMO, the participants were found to have a tendency of “over-reporting” the number of ER visits, which might be caused by outside system usage (3). Yet, among the CDSMP participants without HMO included in the current study, the average number of self-reported ER visits was lower than that from CMS administrative data at all three time points. This may imply outside system ER visits happened less frequently among CMS beneficiaries without HMO who were 65 years or older.

This is one of the first studies that investigated the agreement between self-reported and claim-based administrative data for both chronic conditions and health care utilization variables. It provides an opportunity for us to investigate the correlation between reporting errors for these two types of measures. The agreement between the two data sources for health care utilization and that for chronic conditions were not significantly associated with each other (data not shown). This suggests that the reporting or coding errors were generally distributed randomly, not all happened in a particular subgroup of the study participants. Neither of the two previous studies that reported the concordance between different data sources for both chronic disease diagnosis and health care utilization examined whether the discordant cases were common for chronic conditions and health care utilization (12, 13).

The results of this study are an important contribution to understanding concordance between self-reported and claims-based chronic conditions and utilization of services for older Americans, but need to be interpreted in light of a few limitations. First, the retrospective active consenting process limited the number of CDSMP participants available for linkage to Medicare Administrative Claims Records and analyzed of this study. The consenting process also implies that the specific population studied cannot be assumed to be representative of the general population. In particular, Table 1 revealed that the consented participants had significantly higher number of co-morbidities, physician visits, and hospitalizations at baseline. In addition, those without HMO were better educated and had more physician visits at baseline than those who were enrolled in HMO. This potentially biased final sample further limits the generalizability of our results to general population. Future studies using more broad-based samples with larger sample size would be needed to further inform the controversies on the advantages and disadvantages of difference data sources.

Second, neither Medicare administrative data nor patient self-reported information is a gold standard. Therefore, for the measures with low concordance, it is inconclusive regarding which data source is more accurate. Recall bias is likely a large source of the discordance observed for those measures. Additionally, inaccuracies in self-reported data might be caused by participant’s misunderstanding of the condition or service inquired in the questionnaire. On the other hand, coding errors and inaccurate mapping of the diagnostic and service utilization codes might also be the source of discordance.

Lastly, while evaluating the concordance between two data sources using kappa statistic, although the BI was very or relatively small in most cases, the PI was large for depression and all health care utilization variables. Adjusting for low prevalence of those variables resulted in substantially higher agreement coefficients as measured by PABAK. However, previous methodology research suggested that PABAK values should be interpreted with caution, especially for conditions with low prevalence (28). In those situations, the evaluation and conclusion for the strength of agreement should be judged from multiple aspects, as we did in this study.

In conclusion, the findings of this study expand the existing literature of the concordance between self-reported and medical administrative data for chronic conditions and health care utilization. The findings confirmed the substitutability between self-reports and CMS administrative data for diabetes and hospitalizations in US older population. They also suggest potential substitutability between the two data sources for COPD and ER visits. Finally, it calls for future research on the accuracy of depression and physician visits measures from two data sources.

## Conflict of Interest Statement

Kate Lorig receives royalties from the book used by participants in the CDSMP program. There are no other potential conflicts of interest. Sponsor’s role: Nancy Whitelaw from NCOA contributed to the discussion of this study, reviewed, and edited the manuscript. Kate Lorig receives royalties from the book used by participants in the CDSMP program. There are no other potential conflicts of interest.
